# Bariatric Surgery With or Without Concomitant Laparoscopic Cholecystectomy in Morbidly Obese Patients With Gallbladder Stone Disease: A Prospective Randomized Controlled Pilot Study

**DOI:** 10.1155/jobe/6054585

**Published:** 2026-02-26

**Authors:** Mohamed Atteya Heikal, Ahmed Mohamed Reda Negm, Hosam Mohamed Elghadban, Mahmoud A. Aziz

**Affiliations:** ^1^ General Surgery Department, Faculty of Medicine, Mansoura University, Mansoura, Egypt, mans.edu.eg

**Keywords:** bariatric surgery, concomitant surgery, gallstone disease, laparoscopic cholecystectomy, morbid obesity, randomized controlled trial

## Abstract

**Introduction:**

Imagine a surgeon’s critical decision: Should the gallbladder be removed now, along with the planned bariatric surgery, or risk the complication and necessity of a second surgery later? This clinical dilemma is central to treating morbidly obese patients, who face a high prevalence of gallstone disease exacerbated by rapid postoperative weight loss. The best approach to managing existing gallstones in bariatric candidates remains debated, with debate focusing on whether combining laparoscopic cholecystectomy (LC) with bariatric surgery is both safe and advantageous. In this pilot study, we provide randomized evidence to guide this decision.

**Methods:**

In this prospective randomized controlled pilot study, 58 morbidly obese patients with ultrasound‐confirmed gallstones were randomly assigned to two groups: Group I (*n* = 30) received bariatric surgery and LC; Group II (*n* = 28) had bariatric surgery only, with LC delayed for symptoms. The primary outcomes were clearly defined as operative time, intraoperative complications, and postoperative morbidity, providing a focused measure of safety and efficacy. Secondary outcomes included hospital stay, pain, and follow‐up gallstone symptoms.

**Results:**

Baseline demographics and comorbidities were similar across groups. Operative time was longer in Group I (98.93 ± 11.58 min) than in Group II (75.18 ± 11.26 min, *p* < 0.001). An extra port was used in 20% of Group I patients, compared with none in Group II (*p* = 0.012). No significant differences were observed in bleeding, bile leakage, postoperative complications, or hospital stay. Group I reported higher pain scores (*p* < 0.001). During follow‐up, 79.3% of Group II developed symptomatic gallstones, requiring later cholecystectomy.

**Conclusion:**

Concomitant LC during bariatric surgery in morbidly obese patients with pre‐existing gallstones is demonstrated to be safe and feasible, with acceptable increases in operative time and postoperative pain. The high rate (79.3%) of symptomatic gallstone development in patients who did not undergo concomitant cholecystectomy supports adopting routine concomitant LC to prevent future morbidity, thereby influencing clinical decision‐making and standard practice.

**Trial Registration:** ClinicalTrials.gov: NCT04567890

## 1. Introduction

Morbid obesity is a significant global health problem and is strongly linked with gallbladder disease, especially gallstones (cholelithiasis). In obese people, gallstones are found by ultrasound in about 45%. These individuals are also more likely to develop complications, such as cholecystitis, obstructive jaundice, and pancreatitis [1, 2]. Bariatric surgery is the most effective long‐term treatment for morbid obesity, helping to lower the risks from related health problems [3, 4]. However, rapid weight loss after surgery increases the risk of gallstones. This occurs due to increased bile cholesterol, increased mucin secretion, and weaker gallbladder contractions [5].

While symptomatic gallstones are an accepted indication for laparoscopic cholecystectomy (LC), management of asymptomatic gallstones during BS remains controversial. Some surgeons advocate for prophylactic LC at the time of BS to prevent future complications [6]. In contrast, others recommend a delayed or selective approach due to concerns about increased operative complexity, longer operative times, and potential for additional complications [7, 8].

Debate continues because studies report different rates of developing symptomatic gallstone disease after bariatric surgery, with numbers ranging from 8% to 30% in various groups [9, 10]. This wide range makes it difficult to recommend one management strategy for everyone. Patient age, diet, type of bariatric procedure, and speed of weight loss may all impact the risk of gallstone complications after surgery.

Several meta‐analyses have attempted to address this question, with mixed conclusions. Some suggest that concomitant cholecystectomy is safe and may be beneficial in selected patients [11, 12]. In contrast, others argue that the additional operative burden is not justified by the relatively low incidence of subsequent symptomatic disease [13, 14]. However, most existing studies are retrospective, include heterogeneous patient populations, or lack standardized follow‐up protocols.

Given this clinical equipoise and the paucity of prospective randomized data, we designed this pilot study to compare outcomes between bariatric surgery with concomitant LC versus bariatric surgery alone in morbidly obese patients with pre‐existing gallstones. Our primary objectives were to assess the safety and feasibility of concomitant cholecystectomy and to determine the incidence of symptomatic gallstone disease in patients who did not undergo prophylactic cholecystectomy. We hypothesized that concomitant LC would be safe and feasible, with acceptable increases in operative parameters, and would prevent the need for subsequent interval surgery in a substantial proportion of patients.

## 2. Methods

### 2.1. Study Design and Setting

This prospective, randomized controlled pilot study was conducted at the Department of General Surgery, Mansoura University Hospital, Egypt, from April 2021 to April 2024. The study protocol received Institutional Review Board approval (Faculty of Medicine, Mansoura University, Code: MS/17.09.125). All steps followed institutional research committee guidelines and the Helsinki Declaration. All participants signed written informed consent.

### 2.2. Participants

#### 2.2.1. Inclusion Criteria

Eligible patients met the following conditions:

1. Age 18–60 years.
2. BMI of at least 40 kg/m^2^, or at least 35 kg/m^2^ with additional health problems caused by obesity.
3. Gallstones confirmed by ultrasound.
4. Ready for laparoscopic sleeve gastrectomy according to standard guidelines.
5. ASA Physical Status Class I–III.
6. Able to give informed consent and attend follow‐up visits.

#### 2.2.2. Exclusion Criteria

Patients were excluded if they had the following:

1. Past upper abdominal surgery.
2. Urgent need for cholecystectomy caused by symptomatic gallstones (acute cholecystitis, cholangitis, or gallstone pancreatitis).
3. Stones in the common bile duct seen on imaging. Gallbladder polyps larger than 10 mm or suspected cancer.
4. Serious heart, lung, or liver conditions making surgery unsafe.
5. Were pregnant or planned to be pregnant during the study.
6. Had previous bariatric surgery.
7. Had conditions preventing laparoscopic surgery. Unable to attend follow‐up visits.

### 2.3. Sample Size Calculation

Sample size calculation was based on operative time as the primary feasibility outcome for this pilot study. Using an effect size of 1.139 (derived from institutional pilot data showing a mean difference of 20 min in operative time between bariatric surgery alone versus bariatric surgery with concomitant procedures, with a pooled standard deviation of 17.5 min, yielding Cohen’s *d* = 20/17.5 = 1.14), alpha of 0.05, and power of 80%, a minimum of 28 patients per group was required using G^∗^Power 3.1 software. The choice of sample size was guided by minimizing decision risk and ensuring an acceptable level of uncertainty for surgical planning. Our target was to not miss a clinically significant difference that could impact surgical scheduling and resource allocation, set at a deviation equivalent to our calculated effect size. Our final enrollment of 58 patients (30 in Group I, 28 in Group II) met and slightly exceeded the calculated sample size requirement. The unequal distribution (30 vs. 28) resulted from the simple randomization process but does not compromise statistical power, as both groups meet or exceed the minimum of 28 patients per group.

As a pilot study, our primary objective was to assess the feasibility and safety of performing concomitant LC during bariatric surgery. Operative time is a well‐established feasibility metric that directly reflects procedural complexity, resource utilization, and potential impact on patient outcomes.

### 2.4. Randomization and Allocation

Eligible patients who consented were randomly assigned in a 1:1 ratio to one of two groups. We used Randomizer.org, a validated website that generates random sequences from atmospheric noise, to ensure true random assignment.

To maintain allocation concealment, an independent researcher prepared sequentially numbered, opaque, sealed envelopes (SNOSE) that were not involved in patient recruitment or treatment. Each envelope contained the group assignment, opened only after informed consent was given and baseline assessments completed. This ensured both the physician recruiting the patient and the patient were unaware of the assignment until the time of surgery.

Simple randomization was employed, given the pilot nature of this study with a relatively small sample size (*n* = 58). While stratified or block randomization might have ensured better balance for key prognostic factors, our baseline characteristics analysis (Table 1) demonstrates that the groups were well‐balanced for all measured demographic and clinical variables (age, sex, BMI, comorbidities, stone characteristics), suggesting that simple randomization was adequate for this study. This approach also maintains simplicity and transparency in the randomization process.

**TABLE 1:** Baseline demographic and clinical characteristics.

Characteristic	Group I (*n* = 30)	Group II (*n* = 28)	*p* value
Demographics			
Age (years), mean ± SD	38.4 ± 9.2	39.1 ± 8.7	0.754
Female sex, *n* (%)	22 (73.3)	20 (71.4)	0.871
BMI (kg/m^2^), mean ± SD	46.2 ± 5.3	45.8 ± 4.9	0.768
Comorbidities, *n* (%)			
Type 2 diabetes mellitus	12 (40.0)	10 (35.7)	0.733
Hypertension	11 (36.7)	11 (39.3)	0.835
Dyslipidemia	15 (50.0)	13 (46.4)	0.784
Obstructive sleep apnea	8 (26.7)	8 (28.6)	0.872
GERD	9 (30.0)	9 (32.1)	0.862
Gallstone Characteristics			
Stone size (mm), mean ± SD	12.3 ± 4.2	11.8 ± 3.9	0.628
Multiple stones (≥ 3), *n* (%)	16 (53.3)	14 (50.0)	0.798
GB wall thickness (mm), mean ± SD	3.2 ± 0.8	3.1 ± 0.7	0.609
ASA classification, *n* (%)			
ASA II	18 (60.0)	17 (60.7)	0.957
ASA III	**12 (40.0)**	**11 (39.3)**	

*Note:* The bold values were used to indicate highly statistically significant differences between the two study groups. GERD = gastroesophageal reflux disease; GB = gallbladder.

Abbreviations: ASA, American Society of Anesthesiologists; BMI, body mass index; SD, standard deviation.

### 2.5. Blinding

Given the nature of the surgical intervention, it was not possible to blind the operating surgeons to the allocated procedure. Additionally, postoperative care providers and outcome assessors were not formally blinded to treatment allocation, as this information was necessary for appropriate clinical management, wound assessment, and surgical site monitoring. For pain assessment using the visual analog scale (VAS), patients were asked to rate their pain intensity without being prompted about specific aspects of their surgical procedure; however, patients were aware of their treatment allocation, which may have influenced pain reporting. The lack of blinding for subjective outcomes such as pain is acknowledged as a study limitation and is discussed in the limitations section. However, to mitigate potential assessment bias, identical postoperative protocols were applied across all patient care, ensuring consistent treatment and management irrespective of group allocation.

### 2.6. Operative Technique

All surgical procedures were performed by the same experienced bariatric surgery team (minimum of 100 bariatric procedures and 200 laparoscopic cholecystectomies per surgeon) to minimize intersurgeon variability. Procedures were standardized according to institutional protocols.

### 2.7. Group I: Bariatric Surgery With Concomitant LC

#### 2.7.1. Patient Positioning and Port Placement

Patients were positioned supine with legs apart (French position). Pneumoperitoneum was established using a Veress needle or the open Hassan technique. Five ports were placed: one 12‐mm port placed 15 cm below the xiphoid process and to the left of the midline for the camera, one 15‐mm left upper quadrant port for the stapler, one 5‐mm right upper quadrant port, one 5‐mm left flank port, and one 5‐mm epigastric port (which also served for gallbladder retraction during cholecystectomy).

The greater curvature of the stomach was mobilized from the antrum (approximately 4–6 cm from the pylorus) to the angle of His using a LigaSure device or harmonic scalpel. Short gastric vessels were divided, and the fundus was completely mobilized. A 36‐French bougie was inserted by the anesthesiologist and positioned along the lesser curvature. Gastric resection was performed using sequential firing of endoscopic staplers (typically 4–6 cartridge loads) from the antrum to the angle of His, creating a tubular gastric remnant. The staple line was inspected for hemostasis and integrity. The methylene blue test was performed in selected cases to check for leaks. The resected stomach specimen was placed in the left upper quadrant for later extraction. These standardized steps ensured comparable surgical quality across groups, contributing to the validity of our comparative analysis.

#### 2.7.2. LC

Following completion of the sleeve gastrectomy, cholecystectomy was performed using the critical view of safety technique. The gallbladder was grasped at the fundus and Hartmann’s pouch and retracted cephalad and laterally. The hepatocystic triangle was dissected to achieve the critical view of safety (clear identification of only two structures crossing the hepatocystic triangle: cystic artery and cystic duct, with the lower third of the gallbladder separated from the liver bed). The cystic artery and cystic duct were clipped and divided. The gallbladder was dissected from the liver bed using electrocautery or a harmonic scalpel. Hemostasis was confirmed, and the gallbladder was placed in an extraction bag.

#### 2.7.3. Specimen Extraction

Both the gastric specimen and gallbladder were extracted together through the enlarged 15‐mm left upper quadrant port site or through a small suprapubic incision if specimens were too large. In 20% of cases, an additional 5‐mm port was required for optimal exposure during cholecystectomy.

#### 2.7.4. Drain Placement

An 18‐French silicone drain was placed near the staple line in selected high‐risk cases (at the surgeon’s discretion based on intraoperative findings).

### 2.8. Group II: Bariatric Surgery Alone

**FIGURE 1:**
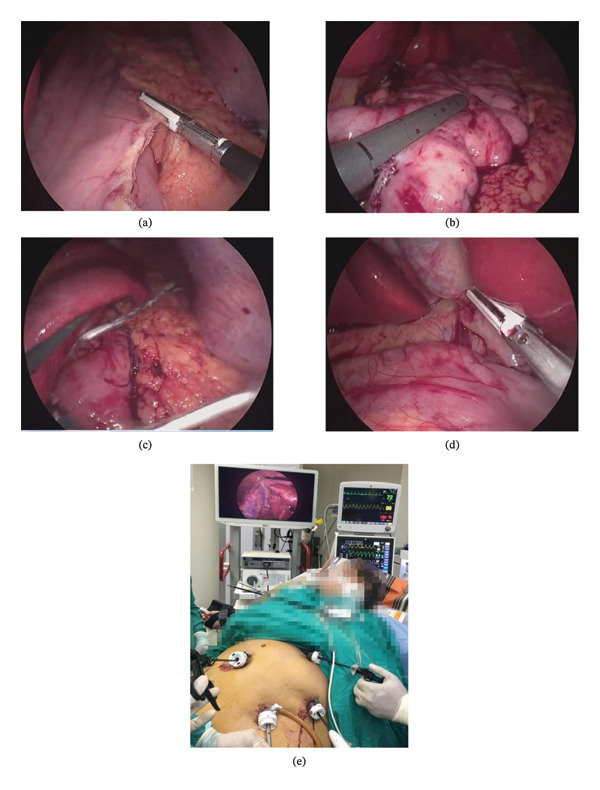
Steps of laparoscopic sleeve gastrectomy with concomitant laparoscopic cholecystectomy. (a) Devascularization of the greater curvature of the stomach using LigaSure. (b) Stapling of stomach. (c) Drain insertion at the staple line after hemostasis. (d) Calot’s triangle dissection using LigaSure. (e) Port design during concomitant laparoscopic cholecystectomy with epigastric port used to grasp fundus of GB and port for camera at midline and two working ports on left and right of camera port as shown at the same time at monitor.

Patients underwent laparoscopic sleeve gastrectomy (Figure 1) using the same technique as described above, but without cholecystectomy. Port placement typically requires four ports (no epigastric port needed). The operative steps were otherwise identical to those in Group I.

### 2.9. Postoperative Management

All patients received standardized postoperative care including: prophylactic antibiotics (single preoperative dose of cephalosporin, extended to 24 h in complicated cases); thromboprophylaxis (low‐molecular‐weight heparin for minimum 10 days); proton pump inhibitors for 3 months; early mobilization (within 6–8 h postoperatively); clear liquid diet starting on postoperative Day 1, advancing to full liquids by Days 2–3; pain management with multimodal analgesia (acetaminophen, NSAIDs, and opioids as needed); and routine upper gastrointestinal contrast study on postoperative Days 2–3 before hospital discharge.

Patients in Group II did not receive routine ursodeoxycholic acid (UDCA) prophylaxis for gallstone disease, as this was not part of our institutional protocol during the study period.

Concise operative protocols, including port placement diagrams and intraoperative decision‐making algorithms, are provided in Supporting File 1.

### 2.10. Statistical Analysis

Statistical analysis was performed using IBM SPSS Statistics Version 26.0 (IBM Corp., Armonk, NY, USA). Continuous variables were first assessed for normality of distribution using the Shapiro–Wilk test and visual inspection of *Q*–*Q* plots and histograms. Variables following normal distribution were expressed as mean ± standard deviation and compared between groups using an independent samples *t*‐test. Non‐normally distributed continuous variables were expressed as median (interquartile range, IQR) and compared using the Mann–Whitney *U* test. Categorical variables were expressed as frequencies and percentages and analyzed using Pearson’s chi‐square test or Fisher’s exact test when expected cell frequencies were less than 5.

Specific statistical tests applied the following: normally distributed continuous variables (age, BMI, operative time, estimated blood loss, hospital stay): independent samples *t*‐test—ordinal variables (pain scores on VAS): Mann–Whitney *U* test—categorical variables (sex, comorbidities, complications, stone characteristics): chi‐square test or Fisher’s exact test—repeated measures (follow‐up pain scores, weight loss): repeated‐measures ANOVA with Bonferroni post hoc correction.

#### 2.10.1. Missing Data

There were no missing data for primary outcomes. All enrolled patients completed the study protocol with full data collection at all time points, eliminating the need for imputation or sensitivity analyses for missing data.

Given the exploratory nature of this pilot study and the limited number of preplanned primary comparisons (operative time, intraoperative complications, and postoperative morbidity), we did not apply formal multiple‐comparison adjustment for the primary outcomes. By choosing unadjusted *p* values, we aim to reassure statisticians about the intent to allow a hypothesis‐generating exploration, recognizing this as a foundation for future confirmatory studies. However, we acknowledge that multiple secondary outcome comparisons increase the risk of Type I error. Therefore, secondary outcome results (pain scores, hospital stay, individual complication types) should be interpreted with appropriate caution, and *p* values between 0.01 and 0.05 should be understood as generating hypotheses for future confirmatory studies rather than definitive evidence. In this manuscript, we report unadjusted *p* values, allowing readers to make their own judgments about statistical significance. A two‐tailed *p* value < 0.05 was considered statistically significant for primary outcomes.

### 2.11. Follow‐Up Protocol and Patient Retention

All patients were followed prospectively according to a standardized protocol with scheduled clinic visits at 1 week, 1 month, 3 months, 6 months, and 12 months postoperatively. At each follow‐up visit, patients underwent the following:

• Clinical examination, including vital signs and surgical wound assessment.
• Symptom assessment using a standardized questionnaire focusing on abdominal pain, nausea, vomiting, jaundice, and biliary‐type symptoms.
• Weight measurement and calculation of excess weight loss.
• Laboratory tests (complete blood count, liver function tests, lipid profile) at 3, 6, and 12 months.
• Abdominal ultrasound at 6 and 12 months (and earlier if symptoms developed).

### 2.12. Patient Retention Strategies

To achieve high follow‐up completeness, we implemented a comprehensive retention strategy that can serve as a replicable model for other centers aiming for similar success. Our strategies included using multiple contact methods; we collected multiple phone numbers (the patient’s mobile and home phones, and at least one family member’s contact), email addresses, and WhatsApp contact information. This approach ensures a reliable communication channel that other healthcare facilities can adopt. Patients received appointment reminders via phone call and text message (WhatsApp) 1 week before and 1 day before each scheduled visit, a tactic that enhances patient engagement and can be easily replicated in different settings.

We offered flexible scheduling, allowing patients who were unable to attend scheduled appointments to choose alternative dates within a 2‐week window of the planned visit. This flexibility can be beneficial for institutions with diverse patient schedules. The hospital provided transportation vouchers or reimbursement for patients with financial constraints, an initiative that can be adapted to increase attendance in various geographic areas.

Telehealth options were arranged for patients unable to attend in person due to distance or temporary circumstances. Through telephone consultations focused on symptom assessment, we facilitated subsequent in‐person visits for imaging and laboratory work. This method supports continuity of care and can inspire similar implementations elsewhere.

A dedicated research coordinator managed appointment scheduling and patient contact, a role that could enhance follow‐up compliance in other research frameworks. Our integrated care approach, which maintains close long‐term relationships through multidisciplinary support group meetings, nutritional counseling sessions, and psychological support services, reinforces patient engagement and can distinguish our program from other bariatric programs aiming for improved retention.

Finally, connecting our strategies to broader adoption highlights the importance of a structured retention strategy that is easily tailored to varying operational environments in healthcare systems worldwide.

### 2.13. Follow‐Up Completeness

No patients were lost to follow‐up during the study period. Complete data were available for all 58 participants at all assessment time points through 12 months. While all patients completed follow‐up assessments at all planned time points, eight patients (13.8%) required rescheduling at least one visit due to personal circumstances, work commitments, or logistical reasons. However, all rescheduled visits were completed within 2 weeks of the initially planned date, and no data were missing for primary or secondary outcomes.

It should be noted that while we report 12‐month follow‐up data in this manuscript, the study is part of an ongoing long‐term follow‐up program, and patients continue to be followed beyond the 12‐month timepoint reported here. The relatively short follow‐up period reported (up to 12 months) may have contributed to our higher retention rate compared to longer term studies.

### 2.14. Outcome Measures

#### 2.14.1. Primary Outcomes

1. Operative time (minutes): Measured from skin incision to skin closure.
2. Intraoperative complications: Including bleeding requiring transfusion, bile duct injury, bowel injury, or conversion to open surgery.
3. Postoperative morbidity: Any complication occurring within 30 days of surgery, classified according to the Clavien–Dindo classification.

#### 2.14.2. Secondary Outcomes

1. Postoperative pain: Assessed using VAS (0–10) at 6, 12, 24, and 48 h postoperatively.
2. Hospital length of stay (days).
3. Intraoperative blood loss (mL).
4. Need for additional ports during surgery.
5. Postoperative complications: including wound infection, bleeding, leak, pulmonary complications, and thromboembolic events.
6. Incidence of symptomatic gallstone disease in Group II during follow‐up.
7. Need for subsequent cholecystectomy in Group II.
8. Weight loss outcomes: percentage of excess weight loss (%EWL) at 6 and 12 months.

#### 2.14.3. Definition of Symptomatic Gallstone Disease

Symptomatic gallstone disease was defined as the presence of one or more of the following: biliary colic: episodic right upper quadrant or epigastric pain lasting ≥ 30 min, often occurring after meals; acute cholecystitis: right upper quadrant pain with fever, leukocytosis, and ultrasonographic findings consistent with acute cholecystitis; choledocholithiasis: jaundice, elevated liver enzymes, and/or dilated common bile duct on imaging; gallstone pancreatitis: acute pancreatitis with evidence of gallstones and no other identifiable cause; persistent dyspepsia with biliary features: recurrent postprandial epigastric or right upper quadrant discomfort, nausea, or food intolerance attributed to gallbladder disease after exclusion of other causes.

All symptomatic patients underwent clinical evaluation, laboratory testing, and imaging (ultrasound and/or CT/MRCP as indicated) to confirm the diagnosis.

## 3. Results

### 3.1. Patient Enrollment and Baseline Characteristics

**FIGURE 2:**
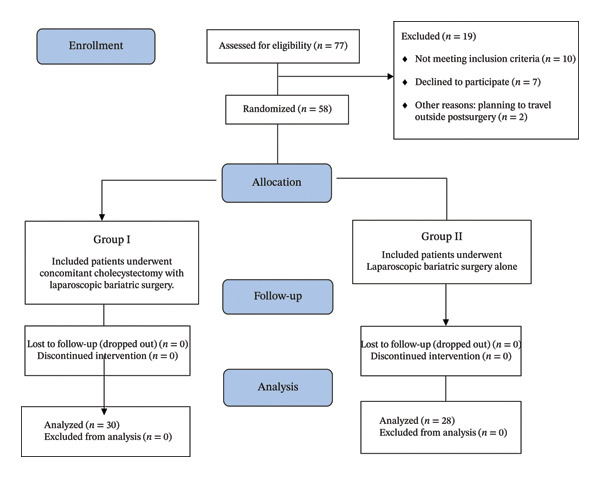
Consort flow diagram showing study design.

Between April 2021 and April 2024, 77 morbidly obese patients with gallstones were screened for eligibility. Of these, 19 patients were excluded: 5 had previous upper abdominal surgery, 3 had symptomatic gallstone disease requiring urgent intervention, 2 had significant comorbidities precluding safe surgery, 7 declined to participate, and 2 planned to travel outside. The remaining 58 patients were randomized: 30 to Group I (bariatric surgery with concomitant LC) and 28 to Group II (bariatric surgery alone). All 58 patients completed the 12‐month follow‐up period with no loss to follow‐up, as shown in Figure 2.

Of 77 patients assessed for eligibility, 58 were randomized after excluding 19 patients. Thirty patients were allocated to concomitant LC with bariatric surgery (Group I) and 28 to bariatric surgery alone (Group II). No patients were lost to follow‐up or discontinued the intervention, and all randomized patients were included in the final analysis.

Baseline demographic and clinical characteristics are presented in Table 1. The two groups were well‐balanced with no significant differences in age, sex distribution, BMI, or prevalence of obesity‐related comorbidities. Mean age was 38.4 ± 9.2 years in Group I and 39.1 ± 8.7 years in Group II (*p* = 0.754). The majority of patients were female (73.3% in Group I and 71.4% in Group II, *p* = 0.871). Mean BMI was similar between groups (46.2 ± 5.3 kg/m^2^ in Group I vs. 45.8 ± 4.9 kg/m^2^ in Group II, *p* = 0.768).

Comorbidities were comparable between groups, including Type 2 diabetes mellitus (40.0% vs. 35.7%, *p* = 0.733), hypertension (36.7% vs. 39.3%, *p* = 0.835), dyslipidemia (50.0% vs. 46.4%, *p* = 0.784), obstructive sleep apnea (26.7% vs. 28.6%, *p* = 0.872), and gastroesophageal reflux disease (30.0% vs. 32.1%, *p* = 0.862).

Gallstone characteristics were also similar between groups. Mean stone size was 12.3 ± 4.2 mm in Group I and 11.8 ± 3.9 mm in Group II (*p* = 0.628). The proportion of patients with multiple stones (≥ 3 stones) was 53.3% in Group I and 50.0% in Group II (*p* = 0.798). Gallbladder wall thickness was 3.2 ± 0.8 mm in Group I and 3.1 ± 0.7 mm in Group II (*p* = 0.609).

### 3.2. Operative Outcomes

Operative outcomes are summarized in Table 2. All procedures were completed laparoscopically with no conversions to open surgery in either group.

**TABLE 2:** Operative outcomes.

Outcome	Group I (*n* = 30)	Group II (*n* = 28)	*p* Value
Operative time (min), mean ± SD	98.93 ± 11.58	75.18 ± 11.26	**< 0.001**
Additional port required, *n* (%)	6 (20.0)	0 (0.0)	0.012
Estimated blood loss (mL), mean ± SD	52.3 ± 18.4	45.7 ± 15.2	0.124
Drain placement, *n* (%)	12 (40.0)	10 (35.7)	0.737
Intraoperative complications, *n* (%)			
Any complication	3 (10.0)	1 (3.6)	0.343
Minor bleeding	2 (6.7)	1 (3.6)	0.607
GB perforation	1 (3.3)	0 (0.0)	0.331
Bile duct injury	0 (0.0)	0 (0.0)	—
Conversion to open	**0 (0.0)**	**0 (0.0)**	—

*Note:* The bold values were used to indicate highly statistically significant differences between the two study groups. GB = gallbladder.

Abbreviation: SD, standard deviation.

### 3.3. Operative Time

Mean operative time was significantly longer in Group I compared to Group II (98.93 ± 11.58 min vs. 75.18 ± 11.26 min, *p* < 0.001), representing a mean increase of 23.75 min (95% CI: 17.84–29.66 min) for the concomitant cholecystectomy. This difference was statistically significant and clinically relevant, reflecting the additional time required for the cholecystectomy procedure.

### 3.4. Port Placement

An additional port (beyond the standard four ports for sleeve gastrectomy) was required in 6 patients (20.0%) in Group I for optimal exposure during cholecystectomy, compared to none in Group II (*p* = 0.012). All additional ports were 5‐mm ports placed in the right upper quadrant or right flank.

### 3.5. Intraoperative Blood Loss

Mean estimated blood loss was slightly higher in Group I (52.3 ± 18.4 mL) compared to Group II (45.7 ± 15.2 mL), but this difference did not reach statistical significance (*p* = 0.124). No patient in either group required a blood transfusion.

### 3.6. Intraoperative Complications

There were no major intraoperative complications in either group. Specifically, there were no bile duct injuries, bowel injuries, major vascular injuries, or splenic injuries. Minor bleeding requiring additional hemostatic measures (but not transfusion) occurred in 2 patients (6.7%) in Group I and 1 patient (3.6%) in Group II (*p* = 0.607). One patient in Group I had a small gallbladder perforation during dissection with bile spillage, which was managed with copious irrigation and did not result in postoperative complications.

### 3.7. Drain Placement

A drain was placed in 12 patients (40.0%) in Group I and 10 patients (35.7%) in Group II (*p* = 0.737), based on the surgeon’s discretion. Drains were typically removed on postoperative Days 2–3 if output was minimal and nonbilious.

### 3.8. Postoperative Outcomes

Postoperative outcomes are presented in Table 3.

**TABLE 3:** Postoperative outcomes.

Outcome	Group I (*n* = 30)	Group II (*n* = 28)	*p* value
Pain scores (VAS 0–10), median (IQR)			
6 h	6 (5–7)	4 (3–5)	**< 0.001**
12 h	5 (4–6)	3 (2–4)	**< 0.001**
24 h	4 (3–5)	2 (2–3)	**< 0.001**
48 h	3 (2–4)	2 (1–2)	0.003
Hospital stay and complications			
Hospital stay (days), mean ± SD	3.2 ± 0.8	3.0 ± 0.7	0.284
Any complication, *n* (%)	4 (13.3)	3 (10.7)	0.764
Wound infection	2 (6.7)	1 (3.6)	0.607
Nausea/vomiting	1 (3.3)	1 (3.6)	0.969
Other minor complications	1 (3.3)	1 (3.6)	0.969
Major complications (CD ≥ III)	0 (0.0)	0 (0.0)	—
30‐day readmission, *n* (%)	1 (3.3)	0 (0.0)	0.331
30‐day reoperation, *n* (%)	0 (0.0)	0 (0.0)	—
Mortality, *n* (%)	**0 (0.0)**	**0 (0.0)**	—

*Note:* The bold values were used to indicate highly statistically significant differences between the two study groups. IQR = interquartile range.

Abbreviations: CD, Clavien–Dindo; SD, standard deviation; VAS, visual analog scale.

#### 3.8.1. Postoperative Pain

Pain scores assessed using the VAS were significantly higher in Group I than in Group II at all measured time points.

At 6 h postoperatively, the median VAS score was 6 (IQR: 5–7) in Group I vs. 4 (IQR: 3–5) in Group II (*p* < 0.001). At 12 h, median VAS was 5 (IQR: 4–6) in Group I vs. 3 (IQR: 2–4) in Group II (*p* < 0.001). By 24 h, median VAS was 4 (IQR: 3–5) in Group I vs. 2 (IQR: 2–3) in Group II (*p* < 0.001). At 48 h, median VAS was 3 (IQR: 2–4) in Group I vs. 2 (IQR: 1–2) in Group II (*p* = 0.003).

Pain scores decreased over time in both groups, but remained consistently higher in Group I throughout the early postoperative period.

#### 3.8.2. Hospital Stay

Mean hospital length of stay was similar between groups: 3.2 ± 0.8 days in Group I and 3.0 ± 0.7 days in Group II (*p* = 0.284). The majority of patients in both groups were discharged on postoperative Day 3 after completion of the routine contrast study and confirmation of adequate oral intake.

#### 3.8.3. Postoperative Complications

Overall 30‐day complication rates were comparable between groups: 13.3% (4/30) in Group I and 10.7% (3/28) in Group II (*p* = 0.764). All complications were minor (Clavien–Dindo Grades I–II) and managed conservatively.

In Group I: wound infection (superficial): 2 patients (6.7%), managed with antibiotics and local wound care; prolonged nausea/vomiting: 1 patient (3.3%), resolved with antiemetics; urinary tract infection: 1 patient (3.3%), treated with antibiotics.

In Group II: wound infection (superficial): 1 patient (3.6%); prolonged nausea/vomiting: 1 patient (3.6%); atelectasis: 1 patient (3.6%), resolved with chest physiotherapy.

There were no cases of staple line leak, postoperative bleeding requiring intervention, bile leak, intra‐abdominal abscess, pulmonary embolism, deep vein thrombosis, or mortality in either group. No patient required reoperation within 30 days.

#### 3.8.4. Readmission

One patient in Group I (3.3%) was readmitted on postoperative Day 8 for persistent nausea and dehydration, managed with intravenous fluids and antiemetics, and discharged after 2 days. No readmissions occurred in Group II during the 30‐day period (*p* = 0.331).

### 3.9. Follow‐Up Outcomes and Symptomatic Gallstone Disease

All 58 patients completed a 12‐month follow‐up with no loss to follow‐up. Weight loss outcomes were similar between groups at all time points. At 12 months, mean %EWL was 68.4 ± 12.3% in Group I and 66.8 ± 11.7% in Group II (*p* = 0.596), indicating that concomitant cholecystectomy did not adversely affect weight loss outcomes.

The most striking finding of this study was the high incidence of symptomatic gallstone disease in Group II (bariatric surgery alone). During the 12‐month follow‐up period, 22 of 28 patients (78.6%) in Group II developed symptomatic gallstone disease requiring subsequent cholecystectomy. When including one additional patient who developed symptoms at 13 months (just after the 12‐month study endpoint), the rate was 23 of 28 patients (82.1%).

Symptom onset occurred at a median of 5.5 months (IQR: 4–8 months) postoperatively. The distribution of symptom onset was as follows: 1–3 months: 4 patients (17.4%); 4–6 months: 11 patients (47.8%); 7–9 months: 5 patients (21.7%); 10–12 months: 2 patients (8.7%); and > 12 months: 1 patient (4.3%).

The peak incidence of symptom development occurred between 4 and 6 months postoperatively, coinciding with the period of most rapid weight loss.

#### 3.9.1. Types of Symptomatic Presentation

The clinical presentations of symptomatic gallstone disease in Group II were as follows: biliary colic (uncomplicated): 15 patients (65.2%); acute cholecystitis: 5 patients (21.7%); choledocholithiasis: 2 patients (8.7%); and gallstone pancreatitis: 1 patient (4.3%).

All 22 patients who developed symptoms during the 12‐month study period underwent subsequent LC. The median time from symptom onset to cholecystectomy was 2.5 weeks (IQR: 1–4 weeks). Twenty of the 22 subsequent cholecystectomies (90.9%) were completed laparoscopically, while 2 cases (9.1%) required conversion to open surgery due to dense adhesions and difficult anatomy. One patient who underwent cholecystectomy for acute cholecystitis had a prolonged hospital stay of 7 days due to surgical site infection.

#### 3.9.2. Subgroup Analysis

We performed exploratory subgroup analysis to identify predictors of symptomatic gallstone development in Group II. Larger stone size (> 10 mm) was associated with a higher risk of symptomatic conversion (88.2% vs. 61.5%, *p* = 0.032). Multiple stones (≥ 3 stones) showed a trend toward a higher symptomatic rate but did not reach statistical significance (85.7% vs. 71.4%, *p* = 0.089). Age, sex, BMI, and rate of weight loss were not significantly associated with symptomatic gallstone development in this small sample.

#### 3.9.3. Patients Remaining Asymptomatic

Only 6 patients (21.4%) in Group II remained asymptomatic throughout the 12‐month follow‐up period. These patients continued to have gallstones on follow‐up ultrasound but did not develop symptoms requiring intervention. They were counseled about the ongoing risk of future symptom development and continued in long‐term follow‐up beyond the study period.

As shown in Table 4, there was no statistically significant difference between the two groups regarding weight loss outcomes. The mean %EWL at 6 months was 52.3 ± 10.2% in Group I and 50.8 ± 9.8% in Group II (*p* = 0.558). Similarly, at 12 months, the mean %EWL was comparable between the two groups (68.4 ± 12.3% vs. 66.8 ± 11.7%, respectively; *p* = 0.596).

**TABLE 4:** Follow‐up outcomes and symptomatic gallstone disease.

Outcome	Group I (*n* = 30)	Group II (*n* = 28)	*p* Value
Weight loss outcomes			
%EWL at 6 months, mean ± SD	52.3 ± 10.2	50.8 ± 9.8	0.558
%EWL at 12 months, mean ± SD	68.4 ± 12.3	66.8 ± 11.7	0.596
Gallstone‐related outcomes			
Symptomatic gallstones, *n* (%)	0 (0.0)	22 (78.6)	**< 0.001**
Time to symptoms (months), median (IQR)	—	5.5 (4–8)	—
Type of presentation, *n* (%)			
Biliary colic	—	15 (65.2)	—
Acute cholecystitis	—	5 (21.7)	—
Choledocholithiasis	—	2 (8.7)	—
Gallstone pancreatitis	—	1 (4.3)	—
Subsequent cholecystectomy			
Required cholecystectomy, *n* (%)	0 (0.0)	22 (78.6)	**< 0.001**
Laparoscopic completion	—	20 (90.9)	—
Conversion to open	—	2 (9.1)	—
Complications of interval surgery	—	**1 (4.5)**	—

*Note:* The bold values were used to indicate highly statistically significant differences between the two study groups. IQR = interquartile range.

Abbreviation: %EWL, percentage of excess weight loss; SD, standard deviation.

In contrast, gallstone‐related outcomes showed a marked difference between the groups. No patients in Group I developed symptomatic gallstones, whereas 22 patients (78.6%) in Group II became symptomatic (*p* < 0.001). The median time to symptom development in Group II was 5.5 months (IQR: 4–8). The most common presentation was biliary colic in 15 patients (65.2%), followed by acute cholecystitis in 5 patients (21.7%), choledocholithiasis in 2 patients (8.7%), and gallstone pancreatitis in 1 patient (4.3%).

Consequently, none of the patients in Group I required subsequent cholecystectomy, while 22 patients (78.6%) in Group II underwent interval cholecystectomy (*p* < 0.001). LC was successfully completed in 20 cases (90.9%), with conversion to open surgery required in 2 cases (9.1%). One patient (4.5%) experienced complications related to interval cholecystectomy.

## 4. Discussion

This prospective randomized controlled pilot study demonstrates that concomitant LC during bariatric surgery in morbidly obese patients with pre‐existing gallstones is safe and feasible, with acceptable increases in operative time and postoperative pain that do not translate into increased morbidity or prolonged hospitalization. Most importantly, our study reveals a remarkably high rate of symptomatic gallstone development (78.6%) in patients who did not undergo prophylactic cholecystectomy, providing compelling evidence in favor of a concomitant approach in this specific patient population.

### 4.1. Safety and Feasibility of Concomitant Cholecystectomy

Our findings confirm that adding cholecystectomy to bariatric surgery can be performed safely without increasing major morbidity. The mean increase in operative time of 23.75 min (approximately 32% longer than bariatric surgery alone) is clinically acceptable and consistent with previous reports [15, 16]. While 20% of patients in the concomitant group required an additional port for optimal exposure, this did not result in increased complications and represents a minor technical consideration.

The slightly higher postoperative pain scores in the concomitant group (median difference of 2 points on VAS in the immediate postoperative period) are expected, given the additional surgical dissection in the right upper quadrant. However, these differences diminished by 48 h and did not result in prolonged hospitalization or increased analgesic requirements beyond the acute postoperative period. Importantly, pain scores in both groups were within acceptable ranges and well‐managed with standard multimodal analgesia protocols.

Critically, there were no differences in major complications, hospital stay, or readmission rates between groups. The absence of bile duct injuries, major bleeding, or other serious complications in the concomitant cholecystectomy group supports the safety of this approach when performed by experienced surgeons using standardized techniques such as the critical view of safety.

### 4.2. High Rate of Symptomatic Gallstone Development

The most striking and clinically significant finding of our study is the 78.6% rate of symptomatic gallstone development in patients who underwent bariatric surgery alone. This rate is substantially higher than the 8%–30% rates reported in most previous studies [9, 10, 17, 18] and warrants careful consideration of several potential contributing factors.

### 4.3. Comparison With Published Literature

Previous studies have reported widely varying rates of symptomatic gallstones after bariatric surgery. A meta‐analysis by Warschkow et al. [18] reported a pooled incidence of 8.9% for symptomatic gallstones after Roux‐en‐Y gastric bypass without prophylactic cholecystectomy. A more recent systematic review by Amorim‐Cruz et al. [17] found rates ranging from 11% to 28%, depending on the study population and follow‐up duration. Our observed rate of 78.6% is markedly higher than these published estimates.

### 4.4. Potential Explanatory Factors

Several factors may explain the high symptomatic conversion rate observed in our study.

#### 4.4.1. Baseline Gallstone Status

Unlike many previous studies that included mixed populations (patients with and without pre‐existing gallstones), our study specifically enrolled only patients with ultrasonographically confirmed gallstones at baseline. Patients with pre‐existing stones undergoing rapid weight loss may represent a particularly high‐risk population for symptom development. Many studies reporting lower rates included patients without baseline stones, which would naturally dilute the symptomatic conversion rate. Our study design specifically addresses how to manage pre‐existing stones, making our findings directly applicable to this clinical scenario.

#### 4.4.2. Dietary and Cultural Factors

Our Egyptian patient population has distinct dietary patterns that may influence gallbladder physiology and symptom manifestation. Traditional Egyptian dietary habits include high consumption of legumes (fava beans, lentils), fats (tahini, olive oil, fried foods), and spices, which can increase gallbladder contractility and, in patients with gallstones, potentially trigger symptoms. The typical Egyptian diet is also characterized by large evening meals and fasting during Ramadan, both of which may affect gallbladder emptying.

Additionally, cultural factors may influence symptom reporting and healthcare‐seeking behavior. In our population, patients may be more likely to report abdominal discomfort and seek medical attention, leading to higher detection rates than in populations where patients might tolerate mild symptoms without seeking care. The close follow‐up relationship maintained in our bariatric program may also have facilitated earlier symptom reporting.

#### 4.4.3. Rapid and Substantial Weight Loss

Our bariatric surgery protocol achieved significant weight loss outcomes, with a mean excess weight loss of 68% at 12 months. Rapid weight loss is a well‐established risk factor for gallstone formation and symptom development [5]. The combination of pre‐existing stones and rapid weight loss may create a particularly high‐risk scenario for symptomatic disease. The peak incidence of symptoms between 4 and 6 months postoperatively in our study coincides with the period of most rapid weight loss, supporting this mechanism.

#### 4.4.4. Definition of “Symptomatic”

Our study used a relatively broad definition of symptomatic gallstone disease, including any biliary‐type pain or significant dyspepsia that could be attributed to gallstones after exclusion of other causes. Some studies use more restrictive definitions, focusing only on severe biliary colic, acute cholecystitis, or complicated gallstone disease requiring urgent intervention. Our more inclusive definition may have captured milder symptomatic presentations that would otherwise be missed or underreported in studies using stricter criteria.

However, we would argue that our definition is clinically appropriate, as even “milder” biliary symptoms significantly impact quality of life and ultimately lead to the decision for cholecystectomy. Indeed, all 22 patients who developed symptoms in our study underwent subsequent cholecystectomy, indicating that symptoms were sufficiently bothersome to warrant surgical intervention.

#### 4.4.5. Intensive Follow‐Up and Systematic Symptom Assessment

Our rigorous follow‐up protocol, with scheduled assessments at multiple time points (1 week, 1 month, 3 months, 6 months, and 12 months) and systematic symptom evaluation at each visit, may have detected symptomatic presentations that would have been missed with less intensive follow‐up schedules. Many retrospective studies or studies with less structured follow‐up may underestimate the true incidence of symptomatic disease, as patients with milder symptoms may not spontaneously report them or seek care unless specifically queried.

The 100% follow‐up completion rate in our study ensures that we did not miss any symptomatic events, whereas studies with significant loss to follow‐up may underestimate symptom incidence if symptomatic patients are more likely to be lost to follow‐up (or conversely, may overestimate if asymptomatic patients are lost).

#### 4.4.6. Absence of UDCA Prophylaxis

Patients in our study did not receive routine UDCA prophylaxis for gallstone disease, as this was not part of our institutional protocol during the study period. Some studies have shown that UDCA prophylaxis can reduce the incidence of gallstone formation and symptomatic disease after bariatric surgery [19]. The absence of UDCA prophylaxis in our Group II patients may have contributed to the high symptomatic rate. However, this also makes our findings more generalizable to settings where UDCA prophylaxis is not routinely used.

## 5. Clinical Implications

Regardless of the specific factors contributing to our high symptomatic conversion rate, this finding has important clinical implications. It suggests that in patients with pre‐existing gallstones undergoing bariatric surgery—particularly in populations with similar demographic, dietary, and cultural characteristics to our Egyptian cohort—the risk of developing symptomatic gallstones requiring subsequent surgery may be substantially higher than previously reported in the literature.

Our data provide strong evidence supporting routine concomitant cholecystectomy in this specific patient population. The modest increases in operative time (24 min) and immediate postoperative pain are clearly outweighed by the prevention of symptomatic disease in nearly 80% of patients and the avoidance of interval surgery with its associated risks, costs, and patient burden.

### 5.1. Cost‐Effectiveness Considerations

While formal cost‐effectiveness analysis was beyond the scope of this pilot study, our findings have important economic implications. Performing concomitant cholecystectomy involves a one‐time increase in operative time and resources. In contrast, a delayed approach results in 78.6% of patients requiring a second surgery, with associated costs including preoperative evaluation, operating room time, anesthesia, hospital stay, and postoperative care. Additionally, 2 of the 22 interval cholecystectomies (9.1%) in our study required conversion to open surgery due to adhesions and difficult anatomy, which would not have been necessary if cholecystectomy had been performed at the initial bariatric procedure.

Previous cost‐effectiveness analyses have suggested that concomitant cholecystectomy may be cost‐effective when the risk of symptomatic disease exceeds 20%–30% [20]. Our observed rate of 78.6% far exceeds this threshold, strongly suggesting that concomitant cholecystectomy would be cost‐effective in this population.

### 5.2. Comparison With Previous Studies

Our findings are consistent with some previous studies supporting concomitant cholecystectomy. Allatif et al. [21] reported that concomitant cholecystectomy in bariatric patients was safe and feasible, with no increase in major complications. Elgohary et al. [22] found that concomitant cholecystectomy was associated with longer operative time but similar complication rates compared to delayed cholecystectomy.

However, our study differs from many previous reports in several important ways. First, our prospective randomized design provides higher‐quality evidence than the retrospective studies that comprise much of the existing literature. Second, our specific focus on patients with documented baseline gallstones addresses a clinically relevant and common scenario. Third, our intensive follow‐up protocol with 100% retention provides more reliable estimates of symptomatic disease incidence than studies with significant loss to follow‐up.

Our findings contrast with the meta‐analysis by Warschkow et al. [18], which concluded that concomitant cholecystectomy was not warranted given the low rates of subsequent symptomatic disease. However, that meta‐analysis included predominantly retrospective studies with heterogeneous populations and variable follow‐up protocols. Our prospective randomized data in a well‐defined population provide new evidence that may warrant reconsideration of this conclusion, at least for specific patient populations. According to current guidelines from the American Society for Metabolic and Bariatric Surgery, routine concomitant cholecystectomy is not universally endorsed due to concerns over increased operative time and potential complications without clear evidence of benefit in asymptomatic patients. By presenting substantial evidence of a high incidence of symptomatic gallstone development in our study cohort, we suggest that these guidelines could be re‐evaluated, particularly for patients with baseline gallstones and similar demographic and cultural characteristics. This reiterates the importance of considering patient‐specific factors in surgical planning to prevent future morbidity.

### 5.3. Population‐Specific Considerations and Generalizability

An important consideration is whether our findings are generalizable to other populations or are specific to our Egyptian cohort. The potential influence of dietary and cultural factors on our high symptomatic rate suggests that similar rates might be observed in populations with comparable characteristics, while different rates might occur in populations with different dietary patterns and cultural contexts.

We believe our findings are most directly applicable to: Middle Eastern and North African populations with similar dietary patterns; populations with high consumption of traditional high‐fat diets; settings where intensive postoperative follow‐up is maintained; patients with documented baseline gallstones (as opposed to mixed populations).

However, even if the symptomatic conversion rate is lower in other populations, any rate substantially above 20%–30% would still favor concomitant cholecystectomy from a cost‐effectiveness and patient burden perspective. We recommend that future studies in diverse populations examine this question to determine whether geographic, dietary, or cultural factors significantly influence outcomes.

### 5.4. Weight Loss Outcomes

An important finding is that concomitant cholecystectomy did not adversely affect weight loss outcomes. The similar %EWL at 6 and 12 months between groups indicates that the additional procedure does not compromise the primary goal of bariatric surgery. This addresses a potential concern that additional surgical trauma or complications might impair weight loss, and provides reassurance that concomitant cholecystectomy can be performed without compromising bariatric outcomes.

### 5.5. Study Limitations

Several limitations of our study should be acknowledged.

#### 5.5.1. Blinding

As discussed in the Methods section, it was not possible to blind surgeons, healthcare providers, or outcome assessors to treatment allocation given the nature of the intervention. The lack of blinding for subjective outcomes, such as pain assessment, may have introduced bias. Patients’ awareness of their treatment allocation could have influenced pain reporting, potentially contributing to higher pain scores in the concomitant cholecystectomy group. Future studies should consider blinding outcome assessors for subjective outcomes where possible and using objective outcome measures less susceptible to assessment bias.

#### 5.5.2. Pilot Study Design and Sample Size

As a pilot study, our sample size was relatively small (*n* = 58) and was powered primarily for feasibility outcomes (operative time) rather than clinical outcomes. While we observed a very high rate of symptomatic gallstones in Group II, the confidence intervals around this estimate are relatively wide given the sample size. Larger multicenter trials are needed to confirm our findings and provide more precise estimates of symptomatic conversion rates in diverse populations.

#### 5.5.3. Single‐Center Study

Our study was conducted at a single tertiary care center with an experienced bariatric surgery team. Results may not be generalizable to centers with less experience or different patient populations. The high level of surgical expertise at our institution may have contributed to the low complication rates observed in both groups.

#### 5.5.4. Follow‐Up Duration

While our 12‐month follow‐up period captures the peak risk period for postbariatric gallstone symptoms, a longer follow‐up would be valuable to determine whether additional patients develop symptoms beyond 12 months. However, the majority of symptomatic presentations in our study occurred within the first 6–9 months, suggesting that a 12‐month follow‐up captures most clinically significant events.

#### 5.5.5. Single Bariatric Procedure

Our study included only laparoscopic sleeve gastrectomy. Results may differ for other bariatric procedures, such as Roux‐en‐Y gastric bypass or adjustable gastric banding, which may have different effects on gallstone disease risk.

#### 5.5.6. Lack of Formal Cost‐Effectiveness Analysis

While our findings suggest that concomitant cholecystectomy would be cost‐effective given the high symptomatic rate, we did not perform a formal cost‐effectiveness analysis. Future studies should include a detailed economic evaluation.

#### 5.5.7. Absence of UDCA Prophylaxis

The lack of UDCA prophylaxis in Group II may have contributed to the high symptomatic rate. Studies comparing concomitant cholecystectomy versus bariatric surgery alone with UDCA prophylaxis would be valuable.

#### 5.5.8. Subgroup Analysis

Our exploratory subgroup analysis suggesting that larger stone size predicts symptomatic conversion should be interpreted with caution, given the small sample size and multiple comparisons. These findings require confirmation in larger studies.

### 5.6. Clinical Recommendations

Based on our findings, we make the following clinical recommendations.

#### 5.6.1. Routine Concomitant Cholecystectomy

In morbidly obese patients with ultrasonographically confirmed gallstones undergoing bariatric surgery, we recommend routine concomitant LC rather than a selective or expectant management approach. The high rate of symptomatic conversion (approaching 80% in our cohort) justifies the modest increases in operative time and immediate postoperative pain.

#### 5.6.2. Patient Counseling

Surgeons should counsel patients with baseline gallstones that: Deferring cholecystectomy carries a high probability (potentially 50%–80%, depending on population and risk factors) of requiring subsequent surgery; concomitant cholecystectomy adds approximately 20–25 min to operative time and may increase immediate postoperative pain but does not increase major complications or hospital stay; and interval cholecystectomy may be more technically challenging due to adhesions and altered anatomy.

#### 5.6.3. Technical Considerations

When performing concomitant cholecystectomy during bariatric surgery: use the critical view of safety technique to minimize risk of bile duct injury; be prepared to place an additional port if needed for optimal exposure; consider performing cholecystectomy before or after sleeve gastrectomy based on surgeon preference and patient anatomy; and ensure adequate hemostasis and irrigation before closure.

#### 5.6.4. Alternative Approaches in Selected Patients

While our data support routine concomitant cholecystectomy, individual patient factors may influence decision‐making: patients with very small stones (< 5 mm) and no symptoms may be candidates for expectant management with close follow‐up; patients with significant comorbidities or technical challenges may benefit from a staged approach if concomitant cholecystectomy would significantly increase operative risk; and shared decision‐making with informed patient consent is essential.

#### 5.6.5. Need for Further Research

Our findings should be confirmed in larger multicenter randomized trials including diverse populations. Future studies should include formal cost‐effectiveness analysis; examine whether dietary, cultural, or geographic factors influence symptomatic conversion rates; compare concomitant cholecystectomy versus bariatric surgery with UDCA prophylaxis; evaluate outcomes for different bariatric procedures; and include longer term follow‐up (> 12 months).

## 6. Conclusion

This prospective randomized controlled pilot study demonstrates that concomitant LC during bariatric surgery in morbidly obese patients with pre‐existing gallstones is safe and feasible. While concomitant LC results in longer operative time (mean increase of 23.75 min) and higher immediate postoperative pain scores, these differences do not translate into increased complications, major morbidity, or prolonged hospital stay.

Critically, 78.6% of patients who underwent bariatric surgery alone developed symptomatic gallstones requiring subsequent cholecystectomy during 12‐month follow‐up. This remarkably high symptomatic conversion rate, substantially exceeding rates reported in many previous studies, provides compelling evidence against a selective or expectant management approach in patients with documented baseline gallstones.

Based on our findings, we recommend routine concomitant cholecystectomy in morbidly obese patients with ultrasonographically confirmed gallstones undergoing bariatric surgery. The modest increases in operative time and postoperative pain are outweighed by the prevention of future gallstone‐related morbidity in the majority of patients, the avoidance of interval surgery with its associated risks and costs, and the high likelihood of symptom development if cholecystectomy is deferred. However, a pressing question remains: Will we allow nearly 80% of our patients to face a second surgery? Addressing this challenge head‐on may drive a shift in practice and ensure our findings remain at the forefront in clinical decision‐making.

While a selective approach (reserving cholecystectomy for symptomatic patients only) remains an option in clinical practice, our data suggest that this strategy will result in the vast majority of patients eventually requiring cholecystectomy, often under less favorable circumstances with increased technical difficulty. Surgeons and patients should be counseled that deferring cholecystectomy in patients with baseline gallstones carries a high probability (approaching 80% in our cohort) of requiring subsequent surgery.

Our findings may be particularly relevant for populations with demographic, dietary, and cultural characteristics similar to those of our Egyptian cohort. The applicability of our results to other populations should be confirmed in future studies, but our data provide strong support for a more aggressive approach to concomitant cholecystectomy than is currently reflected in some clinical practice guidelines.

As a pilot study, our findings require confirmation in larger multicenter trials with diverse populations, longer follow‐up, and formal cost‐effectiveness analysis. Nevertheless, this study provides high‐quality prospective randomized evidence supporting the safety, feasibility, and clinical benefit of routine concomitant cholecystectomy in bariatric patients with pre‐existing gallstones.

## Author Contributions

Mohamed Atteya Heikal: conceptualization, methodology, investigation, data collection, and writing–original draft.

Ahmed Mohamed Reda Negm: conceptualization, methodology, surgery, supervision, and writing–review and editing.

Hosam Mohamed Elghadban: surgery, data analysis, and writing–review and editing.

Mahmoud A. Aziz: methodology, data analysis, statistical analysis, and writing–review and editing.

## Funding

This research received no specific grant from any funding agency in the public, commercial, or not‐for‐profit sectors.

## Disclosure

All authors read and approved the final manuscript. This paper was a part of an MD thesis submitted to the Faculty of Medicine, Mansoura University, in partial fulfillment of the requirements for the MD degree in General Surgery.

## Ethics Statement

This study was approved by the Institutional Review Board of the Faculty of Medicine, Mansoura University and was registered in the Mansoura University IRB database (available at https://irb.mans.edu.eg/IRBsystem.aspx?fn=CurrentResearch%26BibID=368771%26IssueId=373). (Code: MS/17.09.125). All procedures were performed in accordance with the ethical standards of the institutional research committee and with the 1964 Helsinki Declaration and its later amendments. Written informed consent was obtained from all participants.

## Conflicts of Interest

The authors declare no conflicts of interest.

## Supporting Information

Concise operative protocols including port placement diagrams and intraoperative decision‐making algorithms.

## Supporting information


**Supporting Information** Additional supporting information can be found online in the Supporting Information section.

## Data Availability

The datasets used and analyzed during the current study are available from the corresponding author upon reasonable request.
