# Supplementary material for: Bariatric Surgery With or Without Concomitant Laparoscopic Cholecystectomy in Morbidly Obese Patients With Gallbladder Stone Disease: A Prospective Randomized Controlled Pilot Study
Source: J Obes. 2026 Feb 26;2026:6054585. doi: 10.1155/jobe/6054585 (PMC12945468; doi:10.1155/jobe/6054585)
Supplement: Supplementary file 1 — Supporting Information Additional supporting information can be found online in the Supporting Information section. [file JOBE-2026-6054585-s001.docx]

# Supplementary File 1: Concise Operative Protocols

**Manuscript:** Bariatric Surgery With or Without Concomitant Laparoscopic Cholecystectomy in Morbidly Obese Patients With Gallbladder Stone Disease

**Authors:** Mohamed Atteya Heikal, Ahmed Mohamed Reda Negm, Hosam Mohamed Elghadban, Mahmoud Abd-Elaziz

## 1. Preoperative Preparation

Patients were positioned supine in the French position with appropriate arm placement, followed by a 15–30° reverse Trendelenburg tilt after port insertion. All pressure points were padded, and sequential compression devices were applied. The surgeon stood between the patient’s legs, with the assistant on the left and the camera operator and scrub nurse on the right. Prophylactic antibiotics (cefazolin 2 g IV, increased to 3 g for patients >120 kg) were administered within 60 minutes before incision, with redosing as indicated. Thromboprophylaxis included mechanical compression and pharmacologic prophylaxis with low-molecular-weight heparin administered preoperatively.

## 2. Laparoscopic Sleeve Gastrectomy - Concise Protocol

### Step 1: Access and Port Placement

Abdominal access was achieved using the Veress needle at Palmer’s point, with pneumoperitoneum established to 15 mmHg after confirmation of correct intraperitoneal placement. In patients with previous abdominal surgery or suspected adhesions, the open Hasson technique was used. Sleeve gastrectomy was performed using four standard ports, including a left paraumbilical camera port, a subxiphoid port for liver retraction, a right upper quadrant working port for stapler insertion, and a left upper quadrant working port. For concomitant cholecystectomy, the subxiphoid port was also used for gallbladder retraction, with an additional right lateral 5-mm port inserted when necessary to improve exposure.

### Step 2: Abdominal Exploration and Liver Retraction

A systematic inspection of the abdominal cavity was performed to assess liver morphology, identify hiatal hernia, evaluate the stomach and gallbladder, and detect adhesions or anatomic variations. Liver retraction was achieved via the subxiphoid port by gentle elevation of the left lateral segment to expose the gastroesophageal junction and angle of His, taking care to avoid excessive pressure and potential liver injury.

### Step 3: Greater Curvature Mobilization

Mobilization of the greater curvature was initiated 4–6 cm proximal to the pylorus to avoid antral narrowing, with identification of the crow’s foot. The gastrocolic ligament was divided using a vessel-sealing device while preserving the gastroepiploic arcade by staying close to the gastric wall. Dissection was continued cephalad with division of the short gastric vessels to achieve complete fundal and posterior gastric mobilization and expose the left diaphragmatic crus.

### Step 4: Bougie Insertion

A 36-French bougie was inserted under direct vision and advanced along the lesser curvature to the pylorus. Correct alignment was maintained throughout stapling to ensure a straight, uniform gastric sleeve.

### Step 5: Gastric Transection

Gastric transection was initiated 4–6 cm proximal to the pylorus using a 60-mm endoscopic stapler. Stapler firings were aligned parallel to the bougie and proceeded cranially toward the angle of His, maintaining a consistent distance to create a uniform sleeve.

### Step 6: Staple Line Management and completion

The staple line was carefully inspected for hemostasis and integrity, with bleeding controlled using clips and routine absorbable oversewing performed. Selective leak testing was carried out in high-risk cases. The gastric specimen was extracted through an enlarged right upper quadrant port. Drain placement was selective, with a closed-suction drain positioned along the staple line when indicated. A final inspection confirmed hemostasis and the absence of adjacent organ injury, followed by pneumoperitoneum release, fascial closure of ≥10-mm ports, and skin closure.

## 3. Laparoscopic Cholecystectomy - Concise Protocol

### Timing Relative to Sleeve Gastrectomy

Cholecystectomy was performed in Group 2 after laparoscopic sleeve gastrectomy.

Following reverse Trendelenburg positioning with optional left tilt, the gallbladder was exposed by cephalad fundal retraction and lateral traction on Hartmann’s pouch to open the hepatocystic triangle. The peritoneum over the gallbladder neck was incised, adhesions were released, and the cystic structures were progressively exposed. The Critical View of Safety was achieved by complete clearance of the hepatocystic triangle, partial separation of the gallbladder from the liver bed, and confirmation that only the cystic duct and artery entered the gallbladder. The cystic artery and duct were skeletonized, clipped, and divided in a standard fashion. The gallbladder was dissected from the liver bed using electrocautery or energy devices, with careful hemostasis and inspection for bile leakage. The specimen was retrieved in an extraction bag through a 12-mm port. Final inspection confirmed secure clips, hemostasis, and absence of bile leak, with selective drain placement when indicated.

## 4. Postoperative Care

When placed, surgical drains were secured and monitored for volume and character, with removal typically performed once output was <25 mL/day for two consecutive days and non-bilious. Patients were closely monitored in the recovery unit, received supplemental oxygen as needed, multimodal analgesia, antiemetics, and early pharmacologic thromboprophylaxis. Early mobilization, incentive spirometry, and sequential compression devices were emphasized. A clear liquid diet was initiated on postoperative day 1 and advanced as tolerated, with transition to oral analgesia and encouragement of ambulation. Discharge criteria included tolerance of liquids, adequate pain control on oral medications, independent ambulation, and absence of complications. Patients were discharged with structured dietary progression, proton pump inhibitor therapy, bariatric vitamin supplementation, extended thromboprophylaxis, activity restrictions, wound care instructions, and scheduled follow-up visits, with guidance on symptoms warranting urgent medical evaluation.
